# Different rectal toxicity tolerance with and without simultaneous conventionally-fractionated pelvic lymph node treatment in patients receiving hypofractionated prostate radiotherapy

**DOI:** 10.1186/1748-717X-9-129

**Published:** 2014-06-03

**Authors:** Andrew M McDonald, Christopher B Baker, Richard A Popple, Kiran Shekar, Eddy S Yang, Rojymon Jacob, Rex Cardan, Robert Y Kim, John B Fiveash

**Affiliations:** 1 University of Alabama at Birmingham Department of Radiation Oncology, 1700 6th Avenue, South Birmingham, AL 35249, USA; 2 University of Alabama School of Medicine, 1720 2nd Ave S, Birmingham, AL 35294, USA; 3 1924 7th Ave S, Birmingham, AL 35294, USA

**Keywords:** Prostate cancer, Rectal toxicity, Hypofractionation, Pelvic lymph node irradiation

## Abstract

**Purpose:**

To investigate added morbidity associated with the addition of pelvic elective nodal irradiation (ENI) to hypofractionated radiotherapy to the prostate.

**Methods and materials:**

Two-hundred twelve patients, treated with hypofractionated radiotherapy to the prostate between 2004 and 2011, met the inclusion criteria for the analysis. All patients received 70 Gy to the prostate delivered over 28 fractions and 103 (49%) received ENI consisting of 50.4 Gy to the pelvic lymphatics delivered simultaneously in 1.8 Gy fractions. The mean dose-volume histograms were compared between the two subgroups defined by use of ENI, and various dose-volume parameters were analyzed for effect on late lower gastrointestinal (GI) and genitourinary (GU) toxicity.

**Results:**

Acute grade 2 lower GI toxicity occurred in 38 (37%) patients receiving ENI versus 19 (17%) in those who did not (p = 0.001). The Kaplan-Meier estimate of grade ≥ 2 lower GI toxicity at 3 years was 15.3% for patients receiving ENI versus 5.3% for those who did not (p = 0.026). Each rectal isodose volume was increased for patients receiving ENI up to 50 Gy (p ≤ 0.021 for each 5 Gy increment). Across all patients, the absolute V_70_ of the rectum was the only predictor of late GI toxicity. When subgroups, defined by the use of ENI, were analyzed separately, rectal V_70_ was only predictive of late GI toxicity for patients who received ENI. For patients receiving ENI, V_70_ > 3 cc was associated with an increased risk of late GI events.

**Conclusions:**

Elective nodal irradiation increases the rates of acute and late GI toxicity when delivered simultaneously with hypofractioanted prostate radiotherapy. The use of ENI appears to sensitize the rectum to hot spots, therefore we recommend added caution to minimize the volume of rectum receiving 100% of the prescription dose in these patients.

## Introduction

Multiple clinical trials have shown improved biochemical control of clinically localized prostate cancer with increased radiation doses [1-3]. Unfortunately, these dose-escalation regimens have been associated with increased rates of late toxicity, with late rectal toxicity increased two-fold in one early phase III trial [1]. Improvements in treatment planning and delivery, such as intensity-modulated radiotherapy (IMRT) and image-guided radiotherapy (IGRT), have helped to decrease the rate of adverse effects compared to conventional techniques [4,5]. Hypofractionation has also been suggested as a means of achieving a higher biologically effective dose (BED) without leading to increased late toxicity rates.

A growing body of evidence suggests that the biologic response of prostate cancer to larger fraction size exceeds that of the surrounding normal tissues, with the majority of published α/β estimates less than 3 Gy [6,7]. These estimates provide biologic motivation for hypofractionation in order to improve the therapeutic ratio of prostate cancer radiotherapy. By increasing the fraction size and decreasing total dose, an equivalent BED may be delivered to the prostate while decreasing the BED to surrounding normal tissues. Multiple retrospective and phase I/II experiences with moderately hypofractionated regimens (2.5 – 3.0 Gy per fraction) suggest encouraging estimates of biochemical control and toxicity [8-10] and one phase III trial has been reported [11].

Conventionally fractionated pelvic irradiation concurrent with hypofractionated prostate irradiation has been shown to be both feasible and well-tolerated [10,12,13] though any additional morbidity associated these lymphatic treatment volumes has not been quantified. Our preliminary outcomes suggested that the addition of elective nodal irradiation (ENI) was associated with a statistically significant increase in late rectal toxicity [14]. In this work we sought to further explore any association between ENI and increased toxicity. We sought to characterize differences in dose-volume parameters associated with toxicity which could aid future treatment plan generation and evaluation.

## Methods and materials

### Inclusion criteria

The records of all patients receiving external beam radiotherapy for clinically localized prostate cancer since 2004 were reviewed. All patients who received 70 Gy to the prostate delivered over 28 fractions and who had at least 1 year of clinical follow-up were included in the analysis. The study was approved by the University of Alabama at Birmingham institutional review board.

### Simulation and structure definitions

All patients underwent CT simulation in the supine position with a custom immobilization device. Patients were asked to have a full bladder and an empty rectum; if a large quantity of stool was noted in the rectum then patients were resimulated after attempting a bowel movement.

Normal structures contoured for all patients include the femoral heads, bladder, rectum, and bowel space superior to the rectum. The bladder was contoured as a solid organ. The rectum was also contoured as a solid organ from the level of the ischial tuberosities inferiorly to the level of the rectosigmoid junction. Above the level of the rectosigmoid junction the entire peritoneal contents were contoured as one structure.

Three distinct clinical target volumes (CTVs) were defined. The prostate, along with any visible areas of tumor extension, was contoured as the CTV_P_. The entire seminal vesicles were contoured as the CTV_SV_. The CTV_LN_ was generated as a 7 mm uniform expansion around the internal, external, and common iliac vessels. In general, the iliac vessels were countoured to the L5-S1 junction superiorly, though this was somewhat variable. The planned target volumes (PTVs) for the prostate and seminal vesicles, PTV_P_ and PTV_SV_, were generated similarly as a 7 mm expansion around the respective CTV except for posteriorly where the expansion was [4,5] mm. The PTV_LN_ was generated by expanding the CTV_LN_[5-10] mm at the discretion of the treating physician.

### Plan generation and evaluation, treatment delivery, and androgen deprivation

Patients with NCCN low-risk disease were prescribed 70 Gy to the PTV_P_ only. High-risk patients and intermediate risk patients with 10% or greater risk of lymph node involvement by Partin tables [15] were prescribed 70 Gy to the PTV_P_, 56 Gy to the PTV_SV_, and 50.4 Gy to the PTV_LN_. The remaining patients were prescribed 70 Gy to the PTV_P_ and 56 Gy to the PTV_SV_. All prescription doses were delivered simultaneously over 28 fractions.

Treatment plans were generated by either Varian Eclipse or TomoTherapy treatment planning software. Eclipse plans were required to deliver 95% of the prescription dose to the entire PTV and TomoTherapy plans were required to deliver the entire prescription dose to 95% of the PTV. Plans generated in Eclipse utilized either static beam IMRT or volumetric modulated arc therapy (VMAT). A number of dose-volume parameters were used as guidelines for plan generation and the current iteration of our institutional guidelines is included in Table 1. The only constraint weighted higher than PTV coverage was rectal V_70_ < 10 cc based on the work by Kupelian *et al*. with this fractionation regimen [8].

**Table 1:** Current dosimetric guidelines and isodose volumes stratified by ENI

	**Goal**^**1**^	**No ENI**	**ENI**	**p**^**2**^
Rectum		Mean (±SD)		
V_70_	<10 cc^3^	2.1 cc (±1.5 cc)	2.4 cc (± 2.0 cc)	0.369
V_60_	<10 cc^4^	8.3 cc (±4.2 cc)	9.2 cc (±5.0 cc)	0.158
V_50_	<17%^4^	**19.5% (±9.1%)**	**23.0% (±10.6%)**	**0.021**
V_30_	<35%^4^	**53.6% (±20.0%)**	**69.8% (±21.4%)**	**<0.001**
Bladder				
V_60_	<10%^4^	12.1% (±10.1%)	13.4% (±10.8%)	0.197
V_50_	<25%^4^	**20.5% (±15.2%)**	**27.1% (±15.7%)**	**<0.001**
V_30_	<50%^4^	**47.2% (±28.0%)**	**72.9% (±25.5%)**	**<0.001**

^1^Current guidlines; treatment planning goals have become more strict since initially implemented. ^2^Independent samples Kruskal-Wallis test. ^3^Prioritized greater than PTV coverage. ^4^Planning goal but less important than PTV coverage. Bolded text indicates a statistically significant difference.

Treatment was delivered by a TomoTherapy, Varian 2100 series, or Varian TrueBeam linear accelerator. CT-based image guidance with alignment to the prostate-rectum interface was performed prior to the delivery of each fraction.

Patients with intermediate and high-risk disease also received androgen deprivation therapy (ADT) unless they specifically declined this aspect of treatment. ADT consisted of a LHRH analog with an initial short course of anti-androgen. This was begun at least 2 months prior to the start of RT with a total planned duration of 6 months for intermediate-risk patients and 2–3 years for high-risk patients.

### Endpoint definition and follow-up

Patients were scheduled for follow-up visits every 4 months for the first year, every 6 months for an additional 4 years, and annually thereafter. Patients were assessed for toxicity at each visit according to the Common Terminology Criteria for Adverse Events version 4.0 [16]. Only the highest grade toxicity experienced by each patient was taken into account for the analysis. Late toxicity was considered any new toxicity occurring 3 or more months from the completion of RT. Early toxicity which did not resolve within 3 months of the completion of RT was scored as early toxicity only.

### Statistical methods

Patients were stratified based on whether or not they received treatment to the pelvic lymph nodes. Means between the two groups were compared using the independent samples T-test and Kruskal-Wallace independent samples test; frequencies were compared using the Pearson χ2 test. Estimates of late toxicity were calculated by the Kaplan-Meier method and comparison between groups was by the log-rank test. Dose-volume parameters were tested within each group for effect on toxicity by univariate Cox regression modeling. For dose-volume parameters that were statistically significant by Cox regression, cut points were generated by choosing the volume which maximized the discriminatory ability of a receiver-operating characteristic (ROC) curve.

Given that analyzing dose-volume parameters may only predict outcome when above a particular threshold value, ROC curves were generated for each variable which was not statistically significant by Cox regression. A variable was required to generate a *c-*statistic of 0.7 or higher to be considered a good predictor of outcome.

## Results

### Pretreatment and treatment characteristics

A total of 212 patients were included in the analysis. Of the 243 patients treated with this regimen since 2004, 24 were excluded due to inadequate clinical follow-up and 7 treatment plans were unable to be unarchived due to data corruption errors. Median follow-up was 33 months (range 13–96 months). Pelvic lymph nodes were treated in 103 (49%) patients. A table of pretreatment and treatment characteristics is presented as Table 2. Nearly all patients receiving ENI also received concurrent ADT compared to less than half of those not receiving ENI. There was a trend towards a larger prostate volume and a higher proportion of VMAT plans within the group not receiving ENI.

**Table 2:** Pretreatment characteristics

		**No ENI (109)**	**ENI (103)**	***p***
		Frequencies^1^ (%)		
Plan type	Tomotherapy	47 (43%)	47 (46%)	0.081
	IMRT	22 (20%)	28 (28%)	
	Arc	40 (37%)	28 (27%)	
ADT		**44 (40%)**	**98 (95%)**	**<0.001**
		Means^2^ (±SD)		
Age at start of RT		67.5 (±8.5)	67.1 (±7.8)	0.631
Prostate volume (cc)		53.4 (±29.8)	46.2 (±24.3)	0.055
Rectum volume (cc)		92.9 (±53.5)	98.0 (±47.5)	0.467
Bladder volume (cc)		227.7 (±147.4)	206.2 (±164.2)	0.317

^1^Person χ^2^ test. ^2^Independent samples T-test. Bolded text indicates a statistically significant difference.

### Acute toxicity

No patient experienced grade 3 or higher acute toxicity. Acute grade 2 lower GI toxicity occurred in 38 (37%) patients receiving ENI and in 19 (17%) of those that did not (p = 0.001). Grade 2 lower GI toxicity consisted of diarrhea or rectal bleeding that resolved with medical therapy. Acute grade 2 genitourinary toxicity occurred in 54 (52%) of patients receiving ENI and 52 (48%) of those that did not (p = 0.492). Grade 2 genitourinary toxicity consisted of dysuria or hesitancy that resolved with medical therapy. No patient experienced delay or termination of treatment due to acute toxicity.

### Late toxicity

**Figure 1:**
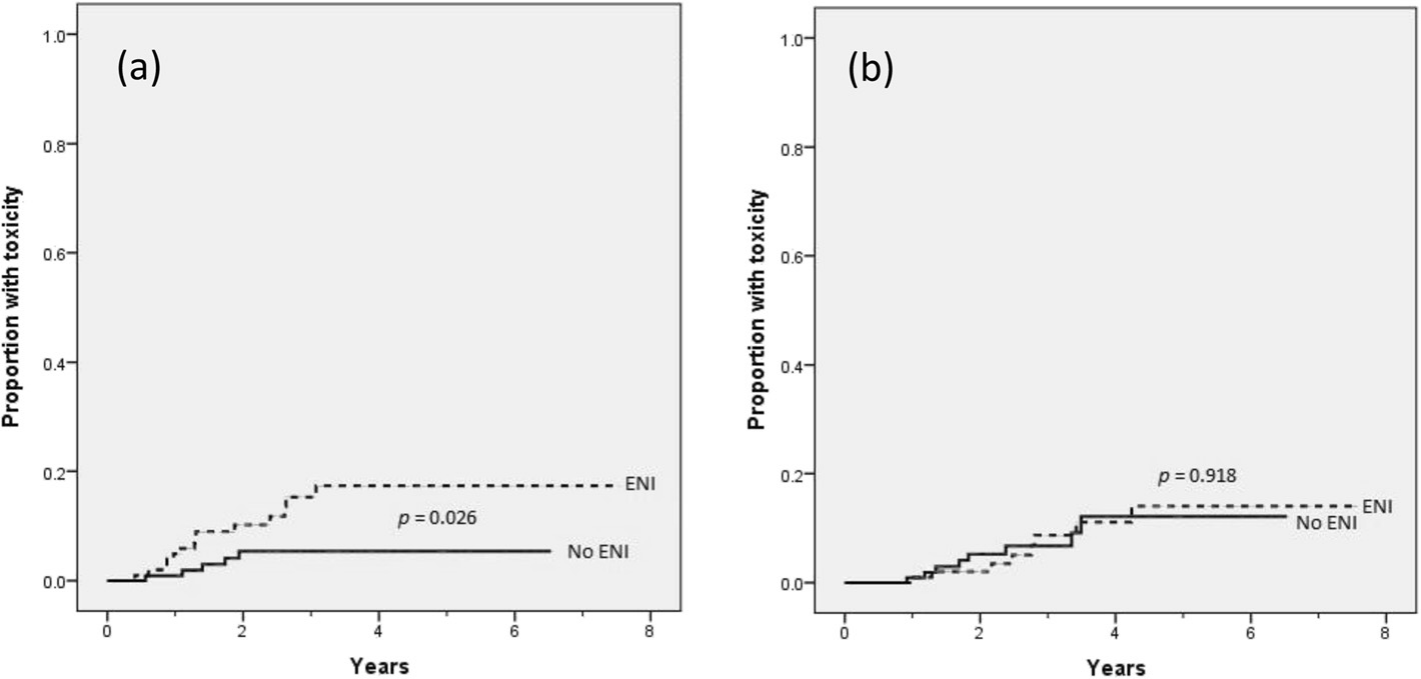
Kaplan-Meier estimates of late grade ≥ 2 lower GI toxicity (a) and late grade 3 GU toxicity (b).

No patient experienced a grade 4 late toxicity event. The Kaplan-Meier estimate of grade ≥ 2 lower GI toxicity (Figure 1a) at 3 years was 15.3% for patients receiving ENI and 5.3% for those who did not (p = 0.026). A Cox regression model yielded a hazard ratio of 3.023 [1.089 – 8.392] associated with the addition ENI. The Kaplan-Meier estimate of grade 3 GU toxicity (Figure 1b) at 3 years was 6.7% for patients receiving ENI and 8.6% for those that did not (p = 0.918). Details of late toxicity events are presented as Table 3.

**Table 3:** Details of late toxicity events stratified by ENI

			**No ENI (109)**	**ENI (103)**
Grade 2 GI events	Diarrhea	Endoscopic evidence of proctitis	1 (1%)	0
		Endoscopy not performed	1 (1%)	1 (1%)
		Endoscopic evidence of proctitis	1 (1%)	4 (4%)
Grade 3 GI events	Rectal bleeding	Required endoscopic laser coagulation procedure	1 (1%)	1 (1%)
		Required blood transfusion	0	1 (1%)
Grade 3 GU events	Urethral stricture requiring dilation		3 (3%)	4 (4%)
	Gross hematuria		4 (4%)	5 (5%)
	Chronic cystitis requiring long-term catheterization		1 (1%)	0

### Dose-volume histogram (DVH) analysis

**Figure 2:**
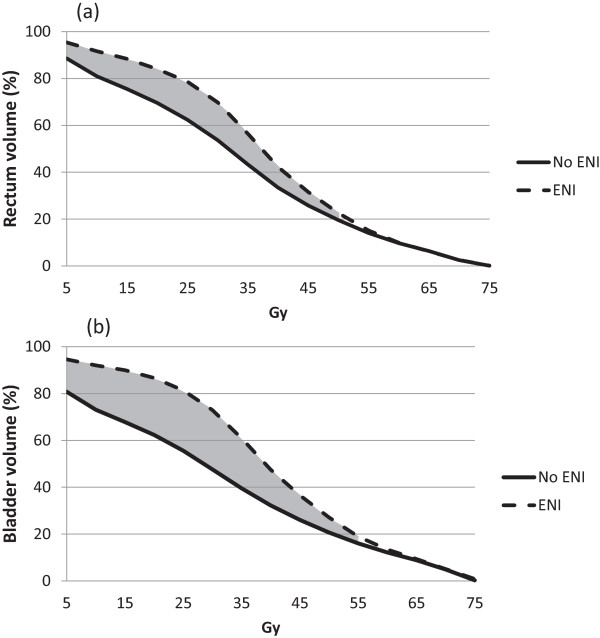
**Mean rectal (a) and bladder (b) dose-volume histograms.** The shaded region indicates a statistically significant difference between the two curves.

The mean bladder and rectal DVHs are presented as Figure 2. Each bladder isodose volume was increased for patients receiving ENI up to the level of 55 Gy (p ≤ 0.027 for each 5 Gy increment). Each rectal isodose volume was increased for patients receiving ENI up to 50 Gy (p ≤ 0.021 for each 5 Gy increment).

Various dose-volume parameters were evaluated for effect on late toxicity (Table 4). ENI and absolute V_70_ were shown to have a statistically significant effect on late lower GI toxicity. No parameter was shown to have an effect on late GU toxicity and no additional parameter was associated with *c* ≥ 0.7 on ROC analysis for either GI or GU toxicity.

**Table 4:** Analysis of dose-volume parameter effect on late toxicity

	**All (212)**		**ENI (103)**		**No ENI (109)**	
	**HR [95% CI]**	***p***	**HR [95% CI]**	***p***	**HR [95% CI]**	***p***
			**GU**			
**Bladder volume**	0.996 [0.991 – 1.001]	0.154	0.996 [0.989 – 1.004]	0.353	0.996 [0.988 – 1.003]	0.242
**Bladder mean dose**	1.000 [1.000 – 1.001]	0.186	1.001 [1.000 – 1.001]	0.059	1.000 [0.999 – 1.001]	0.945
**V**_**40 **_**(percent)**	1.016 [0.996 – 1.036]	0.119	**1.032 [1.003 – 1.062]**	**0.029**	0.964 [0.908 – 1.023]	0.228
**V**_**60 **_**(percent)**	1.021 [0.988 – 1.056]	0.205	1.029 [0.990 – 1.069]	0.150	0.992 [0.828 – 1.188]	0.931
**V**_**60 **_**(percent)**	1.034 [0.974 – 1.098]	0.270	1.044 [0.975 – 1.118]	0.213	1.166 [0.820 – 1.656]	0.955
**V**_**40 **_**(absolute)**	1.007 [0.968 – 1.047]	0.849	1.001 [0.992 – 1.011]	0.806	1.001 [0.980 – 1.022]	0.616
**V**_**60 **_**(absolute)**	1.007 [0.968 – 1.047]	0.733	1.002 [0.953 – 1.053]	0.939	1.017 [0.952 – 1.087]	0.616
**V**_**70 **_**(absolute)**	1.016 [0.944 – 1.094]	0.616	1.007 [0.917 – 1.106]	0.877	1.031 [0.909 – 1.170]	0.631
**ADT**		0.193	-		1.647 [0.805 – 3.367]	0.172
**ENI**	1.517 [0.810 – 2.841]	0.193			-	
**Volume of PTV**_**LN**_	-		1.002 [0.998 – 1.006]	0.245	-	
			**GI**			
**Rectum volume**	1.001 [0.999 – 1.011]	0.081	**1.009 [1.002 – 1.016]**	**0.017**	0.973 [0.934 – 1.014]	0.196
**Rectum mean dose**	1.000 [0.999 – 1.001]	0.401	0.999 [0.999 – 1.000]	0.187	1.000 [0.999 – 1.001]	0.769
**V**_**40 **_**(percent)**	0.993 [0.967 – 1.021]	0.632	0.983 [0.952 – 1.015]	0.287	0.997 [0.938 – 1.059]	0.923
**V**_**60 **_**(percent)**	0.950 [0.847 – 1.065]	0.376	0.968 [0.861 – 1.089]	0.588	0.905 [0.706 – 1.163]	0.439
**V**_**70 **_**(percent)**	1.049 [0.827 – 1.332]	0.692	1.057 [0.814 – 1.374]	0.676	1.033 [0.621 – 1.718]	0.901
**V**_**40 **_**(absolute)**	1.011 [0.996 – 1.026]	0.158	1.011 [0.995 – 1.028]	0.183	0.959 [0.882 – 1.042]	0.319
**V**_**60 **_**(absolute)**	1.049 [0.962 – 1.143]	0.282	1.077 [0.989 – 1.173]	0.087	0.796 [0.598 – 1.060]	0.119
**V**_**70 **_**(absolute)**	**1.249 [1.008 – 1.548]**	**0.042**	**1.314 [1.047 – 1.648]**	**0.018**	0.778 [0.390 – 1.551]	0.476
**ADT**	1.199 [0.719 – 2.000]	0.486			1.548 [0.633 – 3.788]	0.338
**ENI**	**3.023 [1.089 – 8.392]**	**0.034**	-		1.548 [0.633 – 3.788]	0.338
**Volume of PTV**_**LN**_	-		0.999 [0.996 – 1.002]	0.426	-	

Bolded text indicates a statistically significant difference.

**Figure 3:**
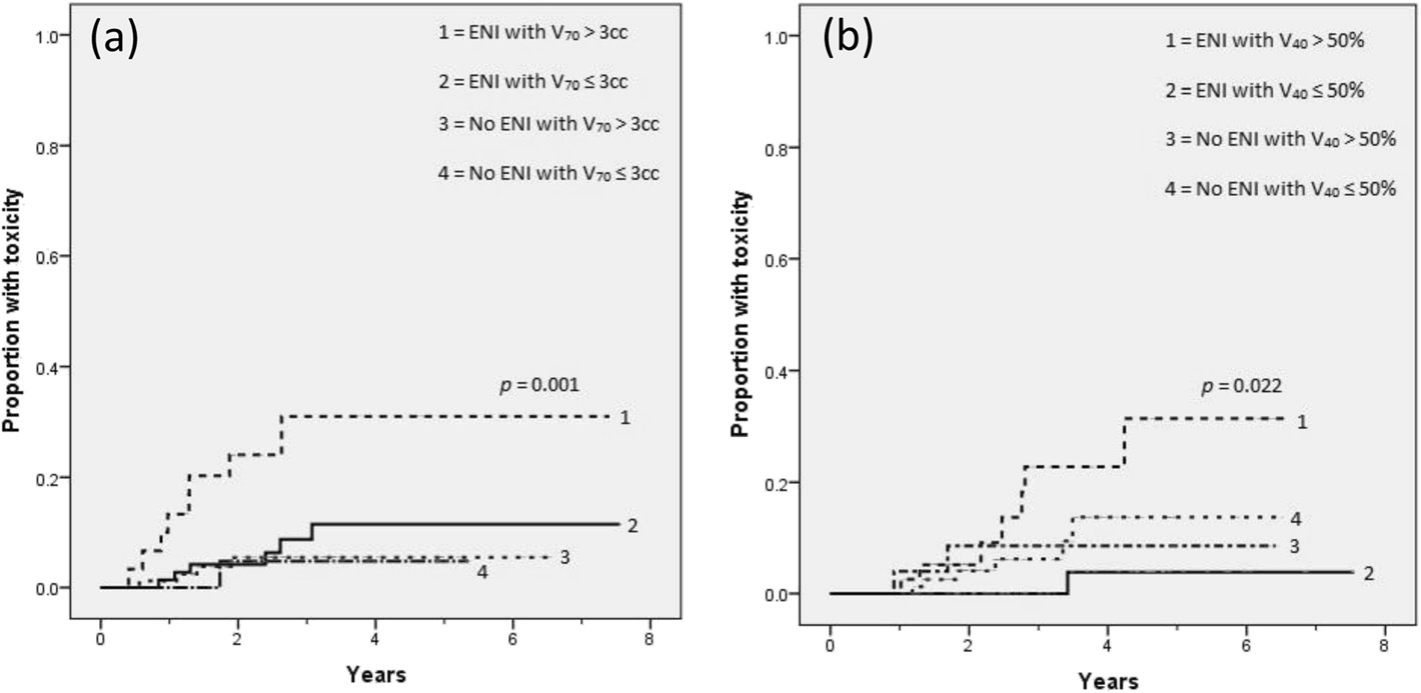
Kaplan-Meier estimates of late grade ≥ 2 lower GI toxicity (a) and late grade 3 GU toxicity (b) stratified by ENI and isodose volume cut-points.

Given the statistically different DVH curves associated with the addition of ENI, we repeated the analysis stratified by the use of ENI. For patients receiving ENI, both rectal volume and the absolute V_70_ of the rectum were predictive of late lower GI toxicity. The percent V_40_ of the bladder was predictive of late GU toxicity for patients receiving ENI. No parameter was predictive of either GI or GU toxicity for patients not receiving ENI. The cut-points generated for rectal V_70_ and bladder V_40_ were 3 cc and 50%, respectively. The Kaplan-Meier plots of rectal and bladder toxicity stratified by these cut-points and ENI are presented as Figure 3.

## Discussion

Data regarding the morbidity of adding conventionally fractionated ENI to hypofractionated prostate radiotherapy remains sparse. A recent review of the literature identified 8 studies with a combined total of 374 patients treated in this manner; however, no study directly addressed to what extent adding ENI increased toxicity [17]. We performed this retrospective study in order to further elucidate the additional toxicity of adding ENI to a hypofractionated regimen and which, if any, dosimetric parameters were predictive of these events. We found the addition of ENI to be associated with increased the rates of both acute and late grade ≥ 2 lower GI toxicity. The rates of GU toxicity were not increased when ENI was utilized.

Comparison of the toxicity rates in our study to the only published large-scale phase III trial of hypofractionation which included treatment to the pelvic lymph nodes in a subset of patients is difficult, as the data has not yet been reported stratified by treatment of the pelvic lymph nodes [11]. Additionally, moderate hematuria requiring cystoscopy and urethral stricture requiring dilatations were scored as grade 2 GU events by Pollack *et al*. whereas we scored such events as grade 3. With this in mind, the rates of late GI and GU toxicity across all patients in our analysis appear similar to those reported in this trial with respect to the revised protocol definition of GU toxicity. This trial did show nodal irradiation to be associated with worse GU outcomes on multivariate analysis whereas we did not observe this effect. The rates of toxicity in our study also fall within the ranges reported by other phase II and retrospective studies [8-10,12,13].

That ENI was associated with increased late rectal toxicity in our study is not altogether surprising given that the whole-pelvic RT plus neoadjuvant ADT (WRPT + NHT) arm of RTOG 94–13 was associated with increased rates of late grade ≥ 3 rectal toxicity [18]. In comparison to the total dose of 70 Gy utilized in this trial, dose-escalation may magnify this effect and IMRT may mitigate it. These questions and others will be explored by the ongoing RTOG 09–24 trial which will further clarify the role of ENI in the conventionally fractionated setting, both in terms of disease control and toxicity.

In terms of dosimetry, the rectal isodose volumes up to the level of 50 Gy were increased for patients receiving ENI. Given the increased lower GI toxicity in patients receiving ENI, we were surprised to find that no isodose volume was predictive of late rectal toxicity where the two DVHs differed. Instead, V_70_ was the only isodose volume which was found to be a predictor of increased toxicity across the entire cohort of patients. When the two groups were analyzed separately, V_70_ was only predictive of toxicity for patients receiving ENI, though the absolute V_70_ was not statistically different between the two groups. Furthermore, the effect of the V_70_ within the ENI group seemed to be driving the effect within the population as a whole. These results suggest that increased volumes of the rectum receiving low and moderate doses decreases the tolerance of the rectum to hot spots in terms of developing clinically apparent toxicity.

That V_70_ was not a predictor of rectal toxicity for patients who did not receive ENI is likely a result of the fact that V_70_ < 8 cc for all patients in our analysis. With 10 cc being the previously reported volume at which increased risk of toxicity for patients not receiving ENI was observed [8], the threshold for observing this effect was simply not met in our study.

Mean bladder isodose volumes were increased in patients receiving ENI up to the level of 55 Gy. Despite this, the rate and type of late GU toxicity was nearly identical between the two groups. With this in mind we expected to find predictors of toxicity above 55 Gy, where the isodose distributions did not differ. However, no dose-volume parameters were found to be predictive of GU toxicity across all patients when analyzed as continuous variables. When the group receiving ENI was analyzed separately, the 40 Gy isodose volume was the only parameter predictive of late toxicity. Given that the V_40_ of the bladder was increased by an average of 15.6% (Figure 2), this likely suggests that patients receiving ENI are simply closer to a threshold region for this parameter. No change in sensitivity to hot spots was recognized for the bladder as was noted for the rectum.

In contrast to the work of Sanguineti *et al*. [19], we did not observe that the addition of ADT increased the risk of late toxicity for patients treated without ENI. However, any synergism between ADT and larger field size, as suggested by the increased rate of rectal toxicity observed in the WPRT + NHT arm of RTOG 94-13 [18], cannot be ruled out. Another limitation of this study includes the inherent biases associated with its retrospective nature. Despite our well-defined inclusion criteria, there was a trend towards a larger mean prostate volume for patients who did not receive ENI, most likely as result of the increased use of neoadjuvant ADT in the ENI group. The effect of this difference between the two groups is likely minimal since daily alignment was to the prostate-rectum interface and the prostate volume did not appear to predict toxicity when analyzed independently. While a median follow-up of 33 months is likely adequate to detect differences in rectal toxicity between the two groups, urinary toxicity events continue to occur for many years after the completion of therapy. Therefore, we cannot rule out differences in GU toxicity which may become apparent with longer follow-up. Lastly, though the addition of ENI increases radiation dose to the small bowel we did not investigate the dosimetry of this structure as a predictor of toxicity within this cohort due to a variety of reasons including significant interfraction variation and a lack of oral contrast at simulation to distinguish large and small bowel, which likely differ in terms of radiosensitivity. We did investigate the size of the elective nodal PTV, which is likely correlated to the volume of small bowel irradiated, as a possible predictor of late toxicity but this was not statistically significant. Additionally, the majority of late toxicity events were manifestations of radiation proctitis.

With these limitations in mind, we recommend taking added caution to minimize the volume of rectum receiving 100% or more of the prescription dose when ENI and ADT are included as part of the treatment plan. While V_70_ ≥ 3 cc was strongly predictive of worse toxicity in patients treated at our institutions, the particular cut point for this isodose volume may vary by institution as a result of differences in methods of treatment planning. As our collective experience with hypofractionated prostate radiotherapy grows, and ongoing clinical trials mature, treatment planning guidelines and normal tissue complication probability modeling should be optimized using multi-institutional outcomes data. Future work should also confirm what appears to be sensitization of the rectal mucosa to hot spots in the setting of larger volumes receiving low and moderate doses in combination with concurrent ADT.

## Conclusions

The addition of conventionally fractionated ENI to hypofractionated prostate radiotherapy increases the rate of late grade ≥ 2 lower GI toxicity when delivered with concurrent ADT. This appears to be due to sensitization of the rectal mucosa to small volumes receiving the prescription dose. We recommend added caution to minimize the volume of rectum receiving 100% or more of the prescription dose when ENI and ADT are included as part of the treatment plan. Achieving V_70_ ≤ 3 cc in our population appears to further reduce the rate of late rectal toxicity.

## Competing interest

The author declares that they have no competing interest.

## Authors’ contribution

AM and JF conceived of the study and drafted the manuscript. CB, RP, and RC performed data collection and extraction of dose-volume histogram information between treatment planning systems. AM and KS performed the statistical analyses. EY, RJ, and RK participated in study design and helped to draft the manuscript. All authors read and approved the final manuscript.
